# Anterior Thalamic Nuclei, but Not Retrosplenial Cortex, Lesions Abolish Latent Inhibition in Rats

**DOI:** 10.1037/bne0000265

**Published:** 2018-10

**Authors:** Andrew J. D. Nelson, Anna L. Powell, Lisa Kinnavane, John P. Aggleton

**Affiliations:** 1 School of Psychology, Cardiff University

**Keywords:** anterior thalamus, attention, nonspatial functions, stimulus preexposure effect, posterior cingulate cortex

## Abstract

The present study examined the effects of excitotoxic lesions in 2 closely related structures, the anterior thalamic nuclei and the retrosplenial cortex, on latent inhibition. Latent inhibition occurs when nonreinforced preexposure to a stimulus retards the subsequent acquisition of conditioned responding to that stimulus. Latent inhibition was assessed in a within-subject procedure with auditory stimuli and food reinforcement. As expected, sham-operated animals were slower to acquire conditioned responding to a stimulus that had previously been experienced without consequence, relative to a non-preexposed stimulus. This latent inhibition effect was absent in rats with excitotoxic lesions in the anterior thalamic nuclei, as these animals conditioned to both stimuli at equivalent rates. The retrosplenial lesions appeared to spare latent inhibition, as these animals displayed a robust stimulus preexposure effect. The demonstration here that anterior thalamic nuclei lesions abolish latent inhibition is consistent with emerging evidence of the importance of these thalamic nuclei for attentional control.

In line with their dense interconnections with the hippocampus, the rodent anterior thalamic nuclei (ATN) are vital for spatial memory and navigation (Aggleton, Hunt, Nagle, & Neave, 1996; Aggleton & Nelson, 2015; Henry, Petrides, St-Laurent, & Sziklas, 2004; Mitchell & Dalrymple-Alford, 2005). The ATN are also reciprocally connected with the prelimbic and anterior cingulate cortices (Mathiasen, Dillingham, Kinnavane, Powell, & Aggleton, 2017; Shibata & Kato, 1993; Shibata & Naito, 2005; Wright, Vann, Erichsen, O’Mara, & Aggleton, 2013), suggesting that the ATN may have cognitive functions beyond the spatial domain. Consistent with this proposal is clinical evidence that pathology or disconnection of the anterior thalamus is associated with anterograde amnesia that extends beyond spatial information, as well as other cognitive disturbances, including executive dysfunction (Ghika-Schmid & Bogousslavsky, 2000; Harding, Halliday, Caine, & Kril, 2000; Little et al., 2010; Nishio et al., 2014). Findings from an array of behavioral paradigms have also implicated the rodent ATN in the processing of nonspatial information (Célérier, Ognard, Decorte, & Beracochea, 2000; Dumont, Amin, & Aggleton, 2014; Law & Smith, 2012; Wolff, Gibb, & Dalrymple-Alford, 2006). Further direct evidence comes from a study that has uncovered a role for the ATN in attentional control: ATN lesions disrupted the formation of attentional sets (impaired intradimensional shifts) but facilitated the learning of discriminations that involved previously irrelevant stimulus dimensions (extradimensional shifts; Wright, Vann, Aggleton, & Nelson, 2015). Remarkably, this pattern of results revealed attentional properties of the ATN that are opposite to those of the prefrontal cortex (Birrell & Brown, 2000). Both clinical and functional imaging evidence has also revealed a similar role in humans (de Bourbon-Teles et al., 2014; Leszczyński & Staudigl, 2016). Taken together, these data suggest that the ATN play a key role in the allocation of attention to those stimuli that are the most reliable predictors of reward (Leszczyński & Staudigl, 2016).

Like the ATN, the retrosplenial cortex (RSC) is heavily implicated in spatial learning and memory (Miller, Vedder, Law, & Smith, 2014; Mitchell, Czajkowski, Zhang, Jeffery, & Nelson, 2018; Vann, Aggleton, & Maguire, 2009), but there is again evidence derived from an array of behavioral paradigms that the RSC may also subserve nonspatial functions (Hindley, Nelson, Aggleton, & Vann, 2014; Keene & Bucci, 2008; Nelson, Hindley, Haddon, Vann, & Aggleton, 2014; Powell et al., 2017; Robinson, Keene, Iaccarino, Duan, & Bucci, 2011; Robinson et al., 2014). Perhaps of particular note is the finding that the RSC may be required for certain forms of cue selection (Keene & Bucci, 2008; Robinson et al., 2011, 2014), as well as an extensive literature implicating the RSC in discriminative avoidance learning, with RSC neurons exhibiting marked training-induced changes in firing patterns (Gabriel, 1993). This neuronal activity in RSC signals both increases in attention to task-relevant cues as well as late-stage cue-based retrieval of learned responses (Gabriel, 1993; Gabriel & Talk, 2001; Smith, Freeman, Nicholson, & Gabriel, 2002; Talk, Stoll, & Gabriel, 2005). Although the RSC and ATN are densely interconnected (Shibata, 1993, 1998; van Groen & Wyss, 1990, 1992, 2003), there is reason to believe that these sites may make distinct contributions to the processing of nonspatial information. For example, unlike ATN damage, RSC lesions spare performance on an attentional set-shifting task that requires animals to track the relevance of stimulus dimensions and subsequently switch from one dimension of reinforced cue to another (Powell et al., 2017); at the same time, these surgeries can produce functional dissociations in the opposite direction (Robinson et al., 2014; Ward-Robinson et al., 2002).

To examine further the involvement of these closely interconnected structures in stimulus processing, the current set of experiments assessed the impact of lesions in the ATN and RSC on latent inhibition. Latent inhibition is demonstrated when nonreinforced preexposure to a stimulus retards subsequent learning about that stimulus (Lubow, 1973; Lubow & Moore, 1959). This latent inhibition effect has been variously ascribed to decreased associability of, or attention to, the preexposed stimulus (Lubow, 1989; Mackintosh, 1975; Pearce & Hall, 1980; Wagner, 1981) or to a performance deficit due to competition between learning from the preexposure and conditioning stages of the procedure (e.g., Grahame, Barnet, Gunther, & Miller, 1994). Despite these debates about the theoretical underpinnings of the effect, latent inhibition has been widely used to examine the neural substrates of learning and attention (Coutureau, Blundell, & Killcross, 2001; Kaye & Pearce, 1987; Nelson, Thur, Marsden, & Cassaday, 2011; Oswald et al., 2002; Weiner, 2003). Disrupted latent inhibition manifests behaviorally as increased conditioning to a stimulus that would normally be treated as irrelevant. Latent inhibition, therefore, provides a direct test of the potential importance of the ATN and RSC for the processing of reward-predictive stimuli.

Consequently, this study assessed the impact of excitotoxic lesions within the ATN (Experiment 1) and RSC (Experiment 2) on latent inhibition. Latent inhibition was assessed in a within-subject conditioning paradigm using auditory stimuli and food reinforcement. Preexposing one of the auditory stimuli results in retarded acquisition of conditioned responding to that stimulus relative to another, non-preexposed auditory stimulus. The key question was, therefore, whether excitotoxic lesions in either the ATN or RSC would disrupt the usual retardation effect.

## Experiment 1: The ATN and Latent Inhibition

### Method

#### Subjects

Experiment 1 involved a cohort of 28 male Lister Hooded rats (Envigo, Bicester, United Kingdom) weighing between 250 and 295 g at the time of surgery. Animals were housed in pairs under diurnal light conditions (14 hr light/10 hr dark). Behavioral testing occurred during the light phase. The rats were handled daily for a week prior to surgery and then randomly assigned to one of two surgical groups: ATN lesions (*n* = 16) or surgical shams (Sham1, *n* = 12). All procedures were carried out in accordance with the U.K. Animals (Scientific Procedures) Act (1986) and EU directive (2010/63/EU), as well as being approved by local ethical committees at Cardiff University. The experiment began approximately a month after surgery. Prior to the current experiment, the rats were tested on spatial alternation in a T maze.

#### Surgical procedures

Anesthesia was induced in all animals using a mixture of oxygen and isoflurane gas before placing them in a stereotaxic frame (David Kopf Instruments, Tujunga, CA), with the incisor bar set at + 5.0 mm to the horizontal plane. Analgesics, Metacam (0.06 ml of a 5-mg/ml solution; Buehringer Ingelheim, Bracknell, United Kingdom) and lidocaine (Xylocaine; AstraZeneca, Luton, United Kingdom), were administered subcutaneously. A midline sagittal incision was made in the scalp, and the skin was retracted to expose the skull. A craniotomy was made above the injection sites. The lesions were made by injecting 0.068 M *N*-methyl-D-aspartate (NMDA; Sigma-Aldrich, Poole, United Kingdom) and 0.063 M ibotenic acid (Sigma-Aldrich, Poole, United Kingdom) solution diluted in phosphate-buffered saline (PBS; 0.1 M, pH 7.4) into two bilateral sites via a 26-gauge 1-μl Hamilton syringe (Bonaduz, Switzerland). The anterior-posterior (AP) and dorsal-ventral (DV) coordinates were measured (in millimeters) from bregma and the mediolateral (ML) coordinates (in millimeters) from the sagittal sinus. The stereotaxic coordinates were as follows: (1) −0.1/0.2 (AP), ± 0.8 (ML), −6.8/6.9 (DV) and (2) −0.2/0.3 (AP), ± 1.5 (ML), −6.2/6.3 (DV). A volume of 0.16 μl of the NMDA/ibotenic acid cocktail was injected at Site 1 and a volume of 0.2 μl at Site 2. Injections were made at a rate of 0.05 μl a minute. After each injection, the needle was left in situ for 5 min. Rats in the surgical control (Sham1) group received identical treatment, except that no neurotoxin was infused. On completion of the surgery, the skin was sutured and clindamycin antibiotic powder (Pfizer, Walton Oaks, United Kingdom) was applied topically, and animals received a subcutaneous injection of glucose saline (5 ml). All animals were given a minimum of 10 days of postoperative recovery before the commencement of T maze behavioral testing.

#### Apparatus

All training was conducted in a set of eight operant boxes (Med Associates, Inc., St. Albans, VT), each measuring 240 mm high × 240 mm deep × 300 mm wide. The boxes were arranged in two rows of four. Each box had two aluminum walls, with a clear Perspex front, back, and ceiling. The grid floor comprised 19 parallel stainless steel bars spaced 16 mm apart. Each operant box was housed in its own sound and light attenuating chamber. Each chamber was illuminated by a 3-W house light located at the top center of the left wall. Auditory stimuli consisted of a 2-kHZ tone and 10-Hz train of clicks, both delivered through ceiling speakers. During training, sucrose pellets (45 mg; P. J. Noyes, Lancaster, NH) were delivered into a recessed food magazine situated in the center of the right-hand wall of the operant box. The magazines were fitted with a pair of infrared detectors that recorded magazine entries. Equipment control and data recording were via a PC equipped with MED-PC software (Med Associates, Inc., St. Albans, VT).

#### Behavioral training

Latent inhibition was examined in a within-subject appetitive conditioning procedure as described by Coutureau et al. (2001). The procedure consisted of three phases.

In each session, the house light was illuminated at the start of every session and remained on for the entire session.

##### Magazine training

All rats received two magazine training sessions, during which a single-pellet reinforcer was delivered into the magazine five times in each 30-min session, according to a random interval 6-min schedule.

##### Preexposure

All rats received three 40-min preexposure sessions, during which one of two auditory stimuli, either a 3-kHz tone or a 10-Hz clicker (counterbalanced across animals), was presented 12 times per session with a mean interstimulus interval of 3 min (random interval schedule). Each stimulus presentation lasted 30 s. No reinforcement was given during these sessions.

##### Conditioning

All rats were then conditioned to both auditory stimuli over six 40-min sessions. In each session, there were two presentations of each of the two auditory stimuli; that is, the preexposed conditioned stimulus (CS) and the non-preexposed stimuli were each presented twice. Stimuli were presented on a random interval 7-min schedule, and the order of presentation was counterbalanced. Stimulus presentation lasted 30 s, and a single reinforcer was delivered into the magazine at the end of each stimulus presentation. Magazine activity was measured during a 30-s period prior to the onset of the CS (pre-CS) as well as during the 30-s CS presentation.

#### Histology

On completion of behavioral testing, rats were deeply anesthetized with sodium pentobarbital (60 mg/kg, intraperitoneal; Euthatal; Merial Animal Health, Harlow, United Kingdom) and transcardially perfused with 0.1 M PBS followed by 4% paraformaldehyde in 0.1 M PBS (PFA). The brains were removed and postfixed in PFA for 4 hr before being transferred to 25% sucrose, in which they remained for at least 12 hr at room temperature, with gentle agitation. A one-in-four series of coronal sections (40 μm) was cut on a freezing microtome and collected on gelatin-coated slides, then subsequently stained using the Nissl stain, cresyl violet.

#### Data analysis

Magazine entry behavior was assessed by examining the total number of entries during the stimulus presentation minus the total number of magazine entries during the 30-s pre-CS period. Data were analyzed in a three-way analysis of variance (ANOVA) with a between-subjects factor of group (ATN vs. Sham1) and within-subjects factors of stimulus (preexposed vs. non-preexposed) and session (1–6). To control for between-subjects variability and to construct error bars appropriate for within-subject comparisons (Figures 2 and 3), the standard error of the mean was calculated using the method proposed by Cousineau (Cousineau, 2005; O’Brien & Cousineau, 2014). These error terms were then corrected according to the method proposed by Morey (2008).[Fig-anchor fig3]

### Results

#### Histology

**Figure 1 fig1:**
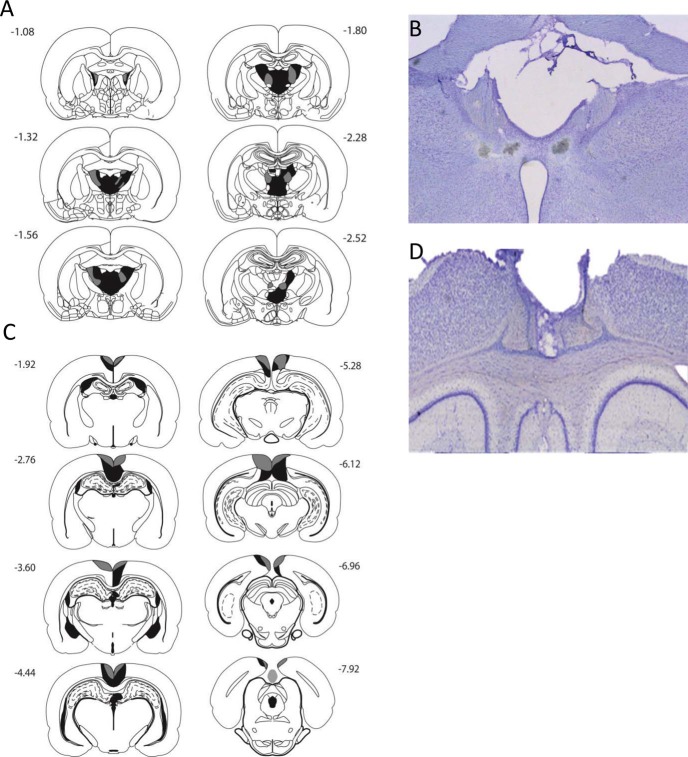
Location and extent of anterior thalamic nuclei (A) and retrosplenial cortex (C) lesions. The coronal reconstructions show the cases with the minimal (dark gray) and maximal (light and dark gray areas) extent of anterior thalamic nuclei (A) and retrosplenial cortex (C) tissue loss, respectively. Photomicrographs from a representative anterior thalamic (C) and retrosplenial cortex (D) lesion. The numbers in A/C indicate the distance (in millimeters) from bregma adapted from *The rat brain in stereotaxic coordinates* (pp. 42–99) by G., Paxinos, & C., Watson, Cambridge, MA: Elsevier Academic Press. Copyright (2005) by Elsevier Academic Press. Reprinted (or adapted) with permission.

The three ATN cases with the smallest anterior thalamic lesions also had evident fornix disruption. These three cases were accordingly excluded. In addition, two shams with unexpected damage to the fornix were also excluded, leaving groups sizes of ATN = 13 and sham controls = 12 (Figure 1A).[Fig-anchor fig1]

Among the 13 ATN cases, there was consistent, bilateral cell loss within the ATN. The lesions were centered in the anteromedial and anteroventral thalamic nuclei, although the anteroventral cell loss was asymmetric in three cases, and all cases involved cell loss in the anterodorsal nucleus. Those animals with essentially complete anterior thalamic lesions also had cell loss in the parataenial nucleus (three cases) and the paraventricular nucleus (four cases). Seven cases also had discrete cell loss in restricted parts of the nucleus reuniens.

#### Behavior

##### Magazine training

Baseline magazine entry activity increased from across the two sessions, *F*(1, 23) = 12.2, *p* < .01, but was unaffected by lesion group (*F* < 1; mean ± *SEM* magazine entries: ATN = 245.3 ± 15.2; Sham1 = 255.3 ± 23.2).

##### Conditioning

Magazine entry behavior in the 30 s prior to the onset of the CS (pre-CS epoch) did not differ by lesion group (*F* < 1). There was an effect of session, *F*(5, 115) = 4.3, *p* < .001, but no effect of CS (*F* < 1) or any interactions between session and lesion (*F*_max_ = 3.4, *p* = .08). Data from the conditioning sessions are, therefore, presented as magazine activity during CS presentation minus magazine activity during the pre-CS period.

**Figure 2 fig2:**
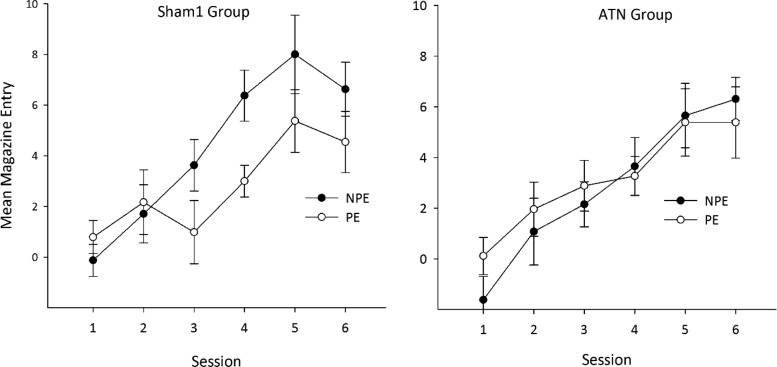
The effect of anterior thalamic nuclei (ATN) lesions on latent inhibition. The mean magazine entries (minus activity in pre–conditioned stimulus period) during presentation of the preexposed stimulus (PE—open symbols) and non-preexposed stimulus (NPE—filled symbols) for sham-operated (left) and ATN lesion (right) animals.

Figure 2 displays the rate of magazine entry behavior for the ATN and Sham1 groups during presentation of the preexposed and non-preexposed CSs across the six sessions of conditioning. As expected, the Sham1 group showed a clear latent inhibition effect in that this group acquired a conditioned response to the preexposed CS at a slower rate relative to the non-preexposed stimulus. In contrast, the latent inhibition effect appeared absent in the ATN lesion group as these animals conditioned to both the preexposed and the non-preexposed stimuli at comparable rates. ANOVA yielded a main effect of session, *F*(5, 115) = 20.54, *p* < .001; no effect of CS, *F*(1, 23) = 2.32, *p* = .14, or lesion, *F*(1, 23) = 1.01, *p* = .33; and no interactions between session and lesion (*F* < 1), or session and CS, *F*(5, 115) = 1.69, *p* = .14, or a three-way interaction between these factors (*F* < 1). However, ANOVA did reveal an interaction between lesion and CS, *F*(1, 23) = 4.99, *p* < .05. Subsequent simple effects confirmed a clear latent inhibition effect in the Sham1 group, *F*(1, 11) = 10.97, *p* < .01, but not in the ATN group (*F* < 1). Furthermore, conditioning to the non-preexposed stimulus differed by lesion group, *F*(1, 23) = 6.47, *p* < .01, but there were no differences between the two groups in the level of conditioning to the preexposed stimulus.[Fig-anchor fig2]

## Experiment 2: RSC and Latent Inhibition

### Method

#### Subjects

Experiment 2 involved a cohort of 28 male Lister Hooded rats (Charles River, Margate, United Kingdom) weighing between 309 and 356 g at the time of surgery. Details of housing and husbandry are the same as described for Experiment 1. Rats were randomly assigned to one of two groups prior to surgery: RSC (*n* = 16) or surgical shams (Sham2, *n* = 12). All procedures were carried out in accordance with the U.K. Animals (Scientific Procedures) Act (1986) and EU directive (2010/63/EU), as well as being approved by local ethical committees at Cardiff University. Behavioral training took place approximately 6 months after surgery. Prior to the current experiment, the animals had been tested on a strategy shift task and matching to place in a T maze (Powell et al., 2017).

#### Surgical procedures

Before the induction of anesthesia, animals received an intraperitoneal injection of atropine (0.06 ml of a 600-μg/ml solution; Martindale Pharma, Brentwood, United Kingdom) and subcutaneous Metacam (0.06 ml of a 5-mg/ml solution; Buehringer Ingelheim, Bracknell, United Kingdom). Rats were then deeply anesthetized (1 ml/kg, intraperitoneal injection) with 6% sodium pentobarbital solution (Ceva Animal Health, Libourne, France), and anesthesia was maintained with isoflurane (∼0.5%) in O_2_ for the duration of the surgery. Rats were placed in a stereotaxic frame (David Kopf Instruments, Tujunga, CA) with the nose-bar set at + 5.0. Lidocaine (Xylocaine; AstraZeneca, Luton, United Kingdom) was administered by subcutaneous injection to the scalp before it was incised and retracted. A bilateral craniotomy, extending from bregma to lambda, exposed the cortex along the midline. Lesions were produced by injecting a solution of 0.09 M NMDA (Sigma-Aldrich, Gillingham, United Kingdom), dissolved in phosphate buffer (pH 7.2), via a 1-μl Hamilton syringe (Bonaduz, Switzerland), at six bilateral sites within the RSC, at a rate of 0.05 μl per minute. After each injection, the needle was left in situ for 5 min.

AP coordinates were measured (in millimeters) from bregma, the ML coordinates (in millimeters) from the sagittal sinus, and the DV coordinates (in millimeters) with reference to the level of the dura. The stereotaxic coordinates at each of the six sites were as follows: (1) −1.8 (AP), ± 0.5 (ML), −1.0 (DV); (2) −2.8 (AP), ± 0.5 (ML), −1.1 (DV); (3) −4.0 (AP), ± 0.5 (ML), −1 (DV); (4) −5.3 (AP), ± 0.5 (ML), −2.5 (DV); (5) −5.3 (AP), ± 0.9 (ML), −1.4 (DV); and (6) −6.6 (AP), ± 0.9 (ML), −1.8 (DV). A volume of 0.25 μl NMDA was injected at Sites 1–3 and 0.27 μl at Sites 4–6.

On completion of the surgery, the skin was sutured and clindamycin antibiotic powder (Pfizer, Walton Oaks, United Kingdom) was applied topically, and animals were rehydrated with glucose saline (5 ml, subcutaneous). Surgical shams received the identical procedure except that the needle was not lowered into the cortex and no NDMA injections were made. All rats received a minimum of 10 days postoperative recovery.

#### Apparatus

The same operant chambers were used as for Experiment 1 except that chocolate flavor food pellets served as reinforcement (45 mg; P. J. Noyes, Lancaster, NH).

#### Behavioral training

All behavioral training was conducted as described for Experiment 1.

### Results

#### Histology

All of the 16 RSC rats displayed considerable cell loss in both the granular and dysgranular subregions. In one rat, there was, however, bilateral sparing of granular RSC rostral to the splenium. A further six animals had minimal sparing of granular A cortex caudal to the splenium. One of these six also had unilateral sparing in the most rostral part of the RSC. Of the 16 RSC animals, 9 had a limited degree of bilateral hippocampal damage (Figure 1B). The extent of this damage varied across animals but was confined within dorsal CA1 in all cases. In 5 of these animals, this bilateral damage affected mainly the medial edge of dorsal CA1, that is, distal CA1. The remaining 4 animals had even more restricted bilateral damage within the same area. As is sometimes seen following lesions in the RSC, around half of the RSC group had ventricular dilatation. In 10 of the 16 animals, there was some minor encroachment into the most caudal part of the anterior cingulate cortex, restricted to the border with the RSC. Following histological analyses, the final group sizes were RSC (*n* = 16) and Sham2 (*n* = 12).

#### Behavior

##### Magazine training

Magazine approach behavior during the two training sessions did not differ by lesion group, and there was no effect of session or a Session × Group interaction (all *F*s < 1; mean ± *SEM* magazine entries: RSC = 250.7 ± 16.95; Sham2 = 266.5 ± 12.4).

##### Conditioning

Magazine approach activity in the 30 s prior to the onset of the CS (pre-CS epoch) did not differ by lesion group (*F* < 1). There was an effect of session, *F*(5, 130) = 4.4, *p* < .001, but no effect of CS (*F* < 1) or any interactions (*F*_max_ = 2.3, *p* = .057).

**Figure 3 fig3:**
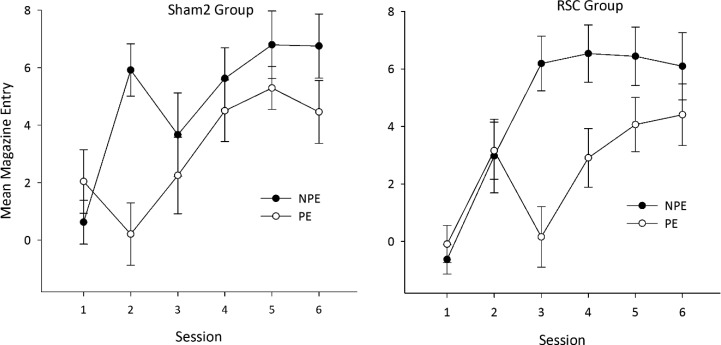
The effect of retrosplenial cortex (RSC) lesions on latent inhibition. The mean magazine entries (minus activity in pre–conditioned stimulus period) during presentation of the preexposed stimulus (PE—open symbols) and non-preexposed stimulus (NPE—filled symbols) for sham-operated (left) and RSC lesion (right) animals.

As is clear from Figure 3, both the Sham2 and RSC groups displayed a stimulus preexposure effect (i.e., lower levels of conditioning to the preexposed CS relative to the non-preexposed stimulus), but the expression of latent inhibition was delayed in the RSC group compared to Sham2 animals. ANOVA supported this description of the data. There was a main effect of CS, *F*(1, 26) = 9.24, *p* < .005, and session, *F*(1, 26) = 17.35, *p* < .001; a Session × CS interaction, *F*(5, 130) = 2.99, *p* < .05; but no effect of lesion (*F* < 1) or interaction between CS and lesion (*F* < 1). However, ANOVA also revealed a three-way interaction between session, CS, and lesion, *F*(5, 130) = 3.78, *p* < .01. Subsequent tests revealed a simple interaction effect between lesion and CS in Session 2, *F*(1, 26) = 10.39, *p* < .01, as the Sham2 group showed a clear latent inhibition effect in Session 2, *F*(1, 11) = 17.04, *p* < .01, but this effect appeared absent in the RSC group (*F* < 1). There was also a simple interaction effect between lesion and CS in Session 3, but here the RSC group showed a clear latent inhibition effect, *F*(1, 15) = 14.89, *p* < .01, whereas there was no difference in responding to the two stimuli in the Sham2 group (*F* < 1).

## Discussion

The current set of experiments examined the impact of damage within the ATN (Experiment 1) and RSC (Experiment 2) on latent inhibition using a within-subject appetitive procedure. As expected, sham-operated rats were slower to acquire conditioned responding to a stimulus that had previously been experienced without consequence relative to a non-preexposed stimulus. This stimulus preexposure effect was absent in the ATN group, as these animals conditioned to the preexposed and non-preexposed stimuli at equivalent rates. In contrast, RSC lesions did not disrupt latent inhibition.

On initial inspection, it might be supposed that the ATN lesions did not disrupt latent inhibition per se but had a more general effect on the acquisition of appetitive Pavlovian conditioning, leading to a nonselective effect on responding irrespective of stimulus preexposure. Although there was no overall effect of lesion, responding to the non-preexposed stimulus was initially lower in the ATN group relative to control animals, but it did reach comparable levels by the end of training. In off-baseline between-subjects assessments of latent inhibition, interpreting a lack of difference in responding between non-preexposed and preexposed animals is problematic when this effect may be mediated by changes in the level of conditioning in the non-preexposed group (Nelson, Thur, & Cassaday, 2012; Weiner & Arad, 2009). However, on-baseline within-subject designs, as employed here, obviate such concerns. Consequently, the critical comparisons are the within-subject differences in the level of responding to the preexposed and non-preexposed stimuli in each group. Moreover, the ATN group exhibited a clear acquisition curve over the six sessions, indicating that the lesions did not produce a generalized deficit in the acquisition of conditioned responding. Indeed, there is considerable evidence derived from a variety of procedures that Pavlovian conditioning is unaffected by ATN damage (Aggleton, Amin, Jenkins, Pearce, & Robinson, 2011; Dumont et al., 2014; Marchand, Faugère, Coutureau, & Wolff, 2014; Ward-Robinson et al., 2002). On this basis, the latent inhibition effect was clearly absent in the ATN group. Despite having been established as an irrelevant stimulus during the preexposure stage, conditioning to the preexposed stimulus proceeded as effectively as to the non-preexposed stimulus in the ATN group, in contrast to the retardation in the acquisition of conditioned responding in the control animals.

The theoretical basis of latent inhibition effect remains moot, as a number of different hypotheses could be put forward to explain ATN lesion-induced disruption of the stimulus preexposure effect. Several prominent theories of latent inhibition posit that the phenomenon arises because associations established between the CS and context during the preexposure phase interfere with either the acquisition or expression of CS → reward associations formed during conditioning (Bouton, 1993; Escobar, Arcediano, & Miller, 2002; Wagner, 1981). Thus, if ATN lesions impair the processing of contextual information, then this might explain the attenuation of latent inhibition seen in the ATN lesion group. The ATN receive prominent inputs from the hippocampal formation (Swanson & Cowan, 1977; Wright, Erichsen, Vann, O’Mara, & Aggleton, 2010), a site critical for the encoding and retrieval of contextual information (Hirsh, 1974; Myers & Gluck, 1994; Smith & Bulkin, 2014). In line with their established role in mediating spatial representations (Jankowski et al., 2013), there is evidence that the ATN may be required for contextual processes (Dumont et al., 2014; Law & Smith, 2012; Marchand et al., 2014). Nevertheless, several factors militate against such an argument. In contrast to the current findings, neither hippocampal nor subicular lesions disrupt latent inhibition (Coutureau, Galani, Gosselin, Majchrzak, & Di Scala, 1999; Oswald et al., 2002; Shohamy, Allen, & Gluck, 2000). In fact, hippocampal inactivation or lesions abolish the context specificity of latent inhibition, leading to its abnormal expression when animals are tested in a novel context (Holt & Maren, 1999; Honey & Good, 1993), a pattern of results that is diametrically opposed to the effects of ATN damage. In any event, ATN lesions do not produce deficits in the processing of the kind of contextual information provided by the experimental chambers used in the current experiments (Dumont et al., 2014). It, therefore, seems unlikely that ATN lesion-induced impairments in the processing of contextual information can provide a satisfactory account of the current findings.

Other theoretical accounts attribute latent inhibition to the conditioning of inattention or to decrements in the associability of, or attention to, the preexposed stimulus (Lubow, 1989; Mackintosh, 1975; Pearce & Hall, 1980). Accordingly, attenuated latent inhibition seen here after ATN lesions may reflect alterations in the attentional processing of the preexposed stimulus. While research into the ATN function has overwhelmingly focused on spatial learning and memory (Aggleton & Nelson, 2015; Jankowski et al., 2013), recent work has uncovered a potential role for these nuclei in attentional control. In an attentional set-shifting paradigm, which measures both attention to stimulus dimensions that reliably predict reinforcement (intradimensional shift) as well as the ability to shift attention to previously irrelevant stimulus dimensions when reward contingencies change (extradimensional shift), ATN lesion animals were slower to learn discriminations involving currently relevant stimulus dimensions but acquired discriminations based on previously irrelevant stimulus dimensions more rapidly than controls (Wright et al., 2015). This pattern of results indicates that in the absence of the ATN, poor predictors of reward usurp attentional control so that animals learn more rapidly about stimuli that have previously been nonpredictive of biologically significant events. The demonstration here that ATN lesions abolished the usual retardation of conditioning to a stimulus that should be treated as irrelevant is consistent with this analysis. The implication is that in the intact brain, the ATN are vital for directing attention to those stimuli that are the best predictors of rewards. Recent human clinical and functional MRI evidence has confirmed that activity in the anterior thalamus tracks the predictiveness of stimulus–stimulus associations (de Bourbon-Teles et al., 2014).

It is striking that the abolition of latent inhibition following lesions in the ATN contrasts with the behavioral effects of damage in related structures, most notably the prefrontal cortex and hippocampus, both sites that are heavily interconnected with the ATN (Wright et al., 2010). Prefrontal manipulations, rather than disrupting the phenomenon, produce the abnormal expression of latent inhibition under conditions (weak preexposure) that do not yield a latent inhibition effect in intact animals (George, Duffaud, Pothuizen, Haddon, & Killcross, 2010; Nelson, Thur, Marsden, & Cassaday, 2010). This dissociation adds to an emerging appreciation that damage to thalamic nuclei can often exert opposing effects on behavior relative to the cortical sites to which they project (Mitchell et al., 2014; Prasad, Abela, & Chudasama, 2017; Wright et al., 2015). The demonstrations that ATN damage does not readily reproduce the behavioral effects of prefrontal lesions contrast with the situation following mediodorsal thalamus lesions, where deficits are often functionally equivalent to the effects of prefrontal damage (Mitchell & Chakraborty, 2013). More broadly, these dissociations highlight how, in the cognitive domain, the function of the thalamus cannot be understood in terms of the thalamus acting as a simple relay of information to the cortex.

Experiment 2 revealed that RSC lesions were without any apparent effect on latent inhibition. Although the expression of latent inhibition appeared to be delayed in these animals relative to their surgical controls, this may reflect variability in the surgical sham group rather than an effect driven by the RSC lesions. In any event, once the latent inhibition effect emerged, the RSC lesion animals exhibited a robust stimulus preexposure effect that was numerically, if not statistically, more enduring than in the controls animals. This null result is perhaps surprising given previous evidence of attenuated neuronal responses to a preexposed stimulus in the RSC (Talk et al., 2005). The present result is unlikely to reflect sparing as the lesions involved tissue along almost the entire AP axis. Furthermore, the lesions were functionally effective as the same animals were impaired on a test of matching to sample in the T maze (Powell et al., 2017). Rather, the lack of an effect suggests that in the absence of the RSC, neuronal activity in other sites may be sufficient to support latent inhibition. Furthermore, the null result is supported by other evidence that retrosplenial damage does not produce attentional deficits (Powell et al., 2017).

A further possibility is that the experimental parameters used in the current study were not sufficiently sensitive to detect retrosplenial involvement in latent inhibition. For example, hippocampal lesion effects on latent inhibition are only apparent when there is a shift in context between preexposure and test (Holt & Maren, 1999; Honey & Good, 1993; Oswald et al., 2002), while frontal effects only emerge when the number of preexposures is limited so that latent inhibition is not present in control animals (George et al., 2010; Joel, Weiner, & Feldon, 1997; Nelson et al., 2010). Thus, a null result following a lesion does not necessarily preclude the involvement of a brain site in latent inhibition. This raises the possibility that RSC lesions potentiate latent inhibition, which is the abnormal expression of the phenomenon under conditions that do not yield latent inhibition in control animals (e.g., weak preexposure, context shift). Given that the RSC is known to be involved in processing contextual information (Miller et al., 2014), a role that the RSC likely fulfills conjointly with the hippocampus, RSC engagement in latent inhibition may be revealed when there is a switch in context between preexposure and test. Electrophysiological evidence that extinguishing the preexposure context reinstates retrosplenial neuronal responses to a preexposed stimulus is consistent with this proposal (Talk et al., 2005). These considerations are, of course, speculative and require empirical testing with appropriate experimental parameters. If, however, RSC lesions potentiate latent inhibition, this would accord with suggestions that the RSC may be required for updating representations as contingencies change.

A further notable feature of the current findings is that ATN and RSC lesions produced dissociable effects on latent inhibition. Thus, despite being heavily interconnected (Shibata, 1993, 1998; van Groen & Wyss, 1990, 1992, 2003), there was no equivalence in the behavioral effects of lesions in these two sites on latent inhibition. One possibility is that differences in the time between surgery and testing as well as the animals’ prior behavioral experience could account for the divergence in findings. This possibility can, of course, not be completely discounted. In contrast to the dissociation found here, lesions in both sites often produce impairments on the same tests of spatial memory, although ATN damage generally results in more severe deficits (Aggleton & Nelson, 2015; Vann et al., 2009). However, in the nonspatial domain, this consistency in the behavioral impact of lesions in these sites is not always found. For example, RSC manipulations impair, while ATN lesions spare, sensory preconditioning (Robinson et al., 2011, 2014; Ward-Robinson et al., 2002). On the other hand, intradimensional and extradimensional set-shifting is unaffected by lesions in the RSC but is sensitive to ATN damage (Powell et al., 2017; Wright et al., 2015). The perhaps unsurprising implication is that, despite their dense interconnectivity, the ATN and RSC make distinct contributions to the processing of nonspatial information.

In summary, pretraining rats with excitotoxic lesions in the ATN, but not the RSC, disrupted the usual retardation in conditioning to a stimulus that has previously been experienced without consequence. These findings add to an emerging literature implicating the rodent ATN in cognitive functions beyond the spatial domain and, in particular, in attentional control (de Bourbon-Teles et al., 2014; Leszczyński & Staudigl, 2016; Wright et al., 2015). The goal of future experiments will be to apply chemogenetic approaches and systems neuroscience analysis to understand how different corticothalamic networks contribute to attention and related executive functions. These endeavors are timely given clinical evidence implicating the rostral thalamus and its frontal connections in disorders that are characterized by marked disturbances of attention (Batty et al., 2015; Mamah et al., 2010; Svatkova et al., 2016; Young, Manaye, Liang, Hicks, & German, 2000).
